# World-Wide Brotherhood

**Published:** 1890-11

**Authors:** 


### World-Wide Brotherhood.

It is a significant fact that medical science knows neither geographical boundaries nor national restraints. Millions of men may be under arms for the preservation of international peace, and yet the devotees of medicine from every land may meet in any domain and under any flag, heartily reciprocate the kindliest courtesies, and labor as brethren in a common effort for the alleviation of human ills, receiving on every hand a heartfelt welcome.

To the honor of the medical profession be it said that the world has never seen such another manifestation of international comity as has just been witnessed at Berlin. The representation from all the principal nations was such as to command profound respect everywhere, and the influence upon the nations represented must have its beneficial influence. The courtesies awarded by the Germans to their French visitors will be sure of a generous response; America will surely be accorded a name and a place upon this planet, and the fact that the centre of medical achievement seemed to be nowhere, and that its circumference is everywhere, will lead the way to more and more generous recognition of *genuine* work and worth regardless of clime or previous condition.

One of the mighty agencies for the unification of the nations will be the unification of their medical men. The international congresses are simply heralding the advance.

